# Effect of reduced z-axis scan coverage on diagnostic performance and radiation dose of neck computed tomography in patients with suspected cervical abscess

**DOI:** 10.1371/journal.pone.0180671

**Published:** 2017-07-05

**Authors:** Jakob Weiss, Michael Maurer, Dominik Ketelsen, Mike Notohamiprodjo, Dominik Zinsser, Julian L. Wichmann, Konstantin Nikolaou, Fabian Bamberg, Ahmed E. Othman

**Affiliations:** 1 Department of Diagnostic and Interventional Radiology, Eberhard Karls University Tuebingen, Tuebingen, Germany; 2 Department of Diagnostic and Interventional Radiology, University Hospital Frankfurt, Frankfurt, Germany; Universidad Francisco de Vitoria, SPAIN

## Abstract

**Purpose:**

To evaluate the effect of reduced z-axis scan coverage on diagnostic performance and radiation dose of neck CT in patients with suspected cervical abscess.

**Methods:**

Fifty-one patients with suspected cervical abscess were included and underwent contrast-enhanced neck CT on a 2^nd^ or 3^rd^ generation dual-source CT system. Image acquisition ranged from the aortic arch to the upper roof of the frontal sinuses (CT_std_). Subsequently, series with reduced z-axis coverage (CT_red_) were reconstructed starting at the aortic arch up to the orbital floor. CT_std_ and CT_red_ were independently assessed by two radiologists for the presence/absence of cervical abscesses and for incidental and alternative findings. In addition, diagnostic accuracy for the depiction of the cervical abscesses was calculated for both readers. Furthermore, DLP (dose-length-product), effective dose (ED) and organ doses were calculated and compared for CT_red_ and CT_std,_ using a commercially available dose management platform.

**Results:**

A total of 41 abscesses and 3 incidental/alternative findings were identified in CT_std_. All abscesses and incidental/alternative findings could also be detected on CT_red_ resulting in a sensitivity and specificity of 1.0 for both readers. DLP, ED and organ doses of the brain, the eye lenses, the red bone marrow and the salivary glands of CT_red_ were significantly lower than for CT_std_ (p<0.001).

**Conclusions:**

Reducing z-axis coverage of neck CT allows for a significant reduction of effective dose and organ doses at similar diagnostic performance as compared to CT_std_.

## Introduction

Cervical abscesses can arise as a complication from various infectious and neoplastic diseases of the naso-pharyngeal area [1,2]. Contrast-enhanced CT imaging of the neck has become a well-established diagnostic tool to assess the primary focus and potential complications, such as venous thrombosis or mediastinitis [3,4]. For a reliable coverage of all possible abscess localizations and related complications, a scan range from the skull base to the aortic arch is recommended [5].

Although cervical abscesses can occur at any age, the highest incidence is found in younger patients [6,7]. Therefore, strategies for CT imaging are necessary in order to reduce the cumulative radiation dose and the risk of potential long-term radiation effects [8,9]. Current approaches for dose reduction focus on examination protocols with automated tube current modulation and automated tube voltage adaption, reduced kVp settings and different iterative reconstruction algorithms [10–12]. Another possibility to effectively reduce dose exposure of CT examinations is to shorten the z-axis scan coverage by excluding those areas, in which the suspected diagnosis is unlikely to occur [13]. Cervical abscesses are most commonly located in the peritonsillar and parapharyngeal region and typical complications involve the adjacent cervical neurovascular pathway and the mediastinum but not the nasopharynx or the skull base [6,14]. Therefore, reduced z-axis scan coverage in the cranial direction might be a possible approach to reduce radiation dose of neck CT without compromising its diagnostic performance for this clinical indication.

Regardless of the chosen dose saving approach, the only readily available dose values to assess for changes in radiation burden are the CTDI_vol_ and the DLP values, indicated by the patient´s protocol [15]. However, these dose values only insufficiently account for size-specific patient characteristics [16,17]. Moreover, the resulting differences in effective dose and specific organ doses can only be approximated in complex calculations or phantom studies, which is not feasible for clinical routine [18,19]. In this context, workflow integrated dose measurement platforms may be helpful to improve patient safety by providing an individual, more detailed dose report, including effective dose and organ doses.

Therefore, the purpose of our study was to evaluate the effect of a reduced z-axis scan coverage on diagnostic performance and radiation dose in contrast-enhanced neck CT (CT_red_) of patients with suspected cervical abscess, using a workflow integrated dose management platform.

## Materials and methods

This retrospective study of routine clinical data was approved by the local ethic committee of the University of Tuebingen, which waived the requirement for written informed consent.

### Patient characteristics

**Fig 1:**
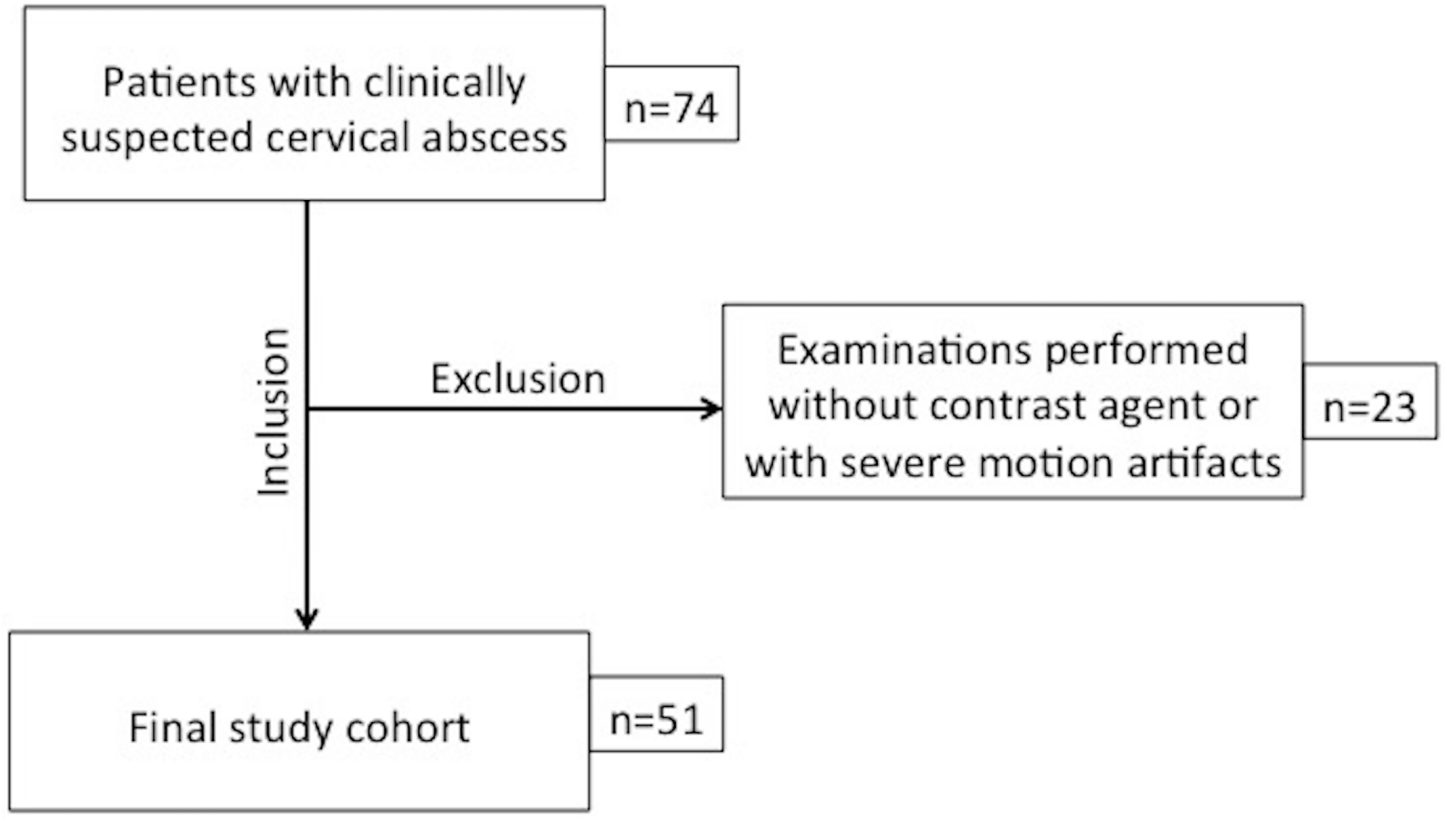
Flowchart of the inclusion and exclusion criteria.

In the time between January 2014 and January 2016 we identified 74 patients who underwent CT examination of the head and neck due to clinically suspected cervical abscesses. Exclusion criteria were examinations performed without contrast agent (n = 19) and examinations with severe motion artifacts (n = 4) leaving a final study cohort of 51 patients. A flowchart of the study design is provided in Fig 1. Further detailed patient demographics are provided in Table 1.

**Table 1:** Patient demographics and acquisition parameters.

Variables		N; Mean±SD
**Patients**		51
**Age**		50.2±16.1 (range: 22–92)
**Sex m:f**		28:23
**BMI**		24.3±4.6
**Clinical Diagnosis**		
***Abscess***		
	peritonsillar	18
	retropharyngeal	15
	parapharyngeal	5
***Tonsillitis***		6
***SCC***	oropharyngeal	5
	hypopharyngeal	2
**Somatom Flash**		
	Single-energy mode: 120 kV	
	Activated automatic attenuation-based tube current modulation (CareDose4D)	
	Reference tube-current time product 200 mAs;	
	Gantry rotation time 0.33 s;	
	Pitch 0.7	
	Collimation 0.6 mm	
**Somatom Force**		
	Dual-energy mode: Tube A 90 kV; Tube B Sn150 kV	
	Activated automatic attenuation-based tube current modulation (CareDose4D)	
	Reference tube-current time product tube A 150 mAs; tube B 115 mAs;	
	Ratio of 120 kV linearly blended images 0.8 (tube A:B)	
	Gantry rotation time 0.25 s;	
	Pitch 0.7	
	Collimation 0.6 mm	

BMI = body mass index; SCC = squamous cell carcinoma

### Acquisition parameters and image reconstruction

All patients underwent contrast-enhanced neck CT with body-weight adapted iodine contrast agent administration (Imeron 400, Bracco, Konstanz, Germany) at a flow rate of 1.5 ml/s using an automated double-syringe power injector (Medrad, Bayer, Germany). Examinations were performed on a 2^nd^ (Somatom Flash, Siemens Healthineers, Forchheim, Germany) or 3^rd^ generation dual-source CT system (Somatom Force, Siemens Healthineers, Forchheim, Germany). For optimal contrast of the cervical soft tissue and vasculature, contrast agent was administered using a double bolus technique. Image acquisition started 3 minutes after the first bolus (80 ml) and 30 seconds after the second bolus (20 ml), respectively, followed by a saline flush. Further detailed acquisition parameters are provided in Table 1.

The standard scan range (CT_std_) started at the aortic arch and included the frontal sinuses completely. From the acquired CT data, images were reconstructed by applying an iterative reconstruction algorithm (Somatom Force; Advanced Modeled Iterative Reconstruction–ADMIRE, strength level 2, Somatom Flash; Sinogram Affirmed Iterative Reconstruction—SAFIRE strength level 3, Siemens, Germany) with a medium hard kernel Bf40 and a slice thickness and increment of 2 mm. In addition, a series with cranially reduced z-axis coverage was reconstructed with the same parameters starting at the aortic arch but terminating just below the orbital floor (CT_red_).

### Image analysis

Image analysis was performed on a dedicated workstation (*syngo*.via, A30A; Siemens Healthineers, Germany) by two independent readers with two (M.M.) and three (J.W.) years of experience in head and neck imaging. Both readers were blinded to the clinical diagnosis. All images were evaluated in a random order for the presence/absence of cervical abscesses and for alternative as well as incidental findings. CT_std_ served as standard of reference to evaluate the diagnostic performance of the CT_red_ reconstructions and to identify any findings that would be missed due to the reduced z-axis coverage.

### Dose measurements

#### Dose management platform

**Fig 2:**
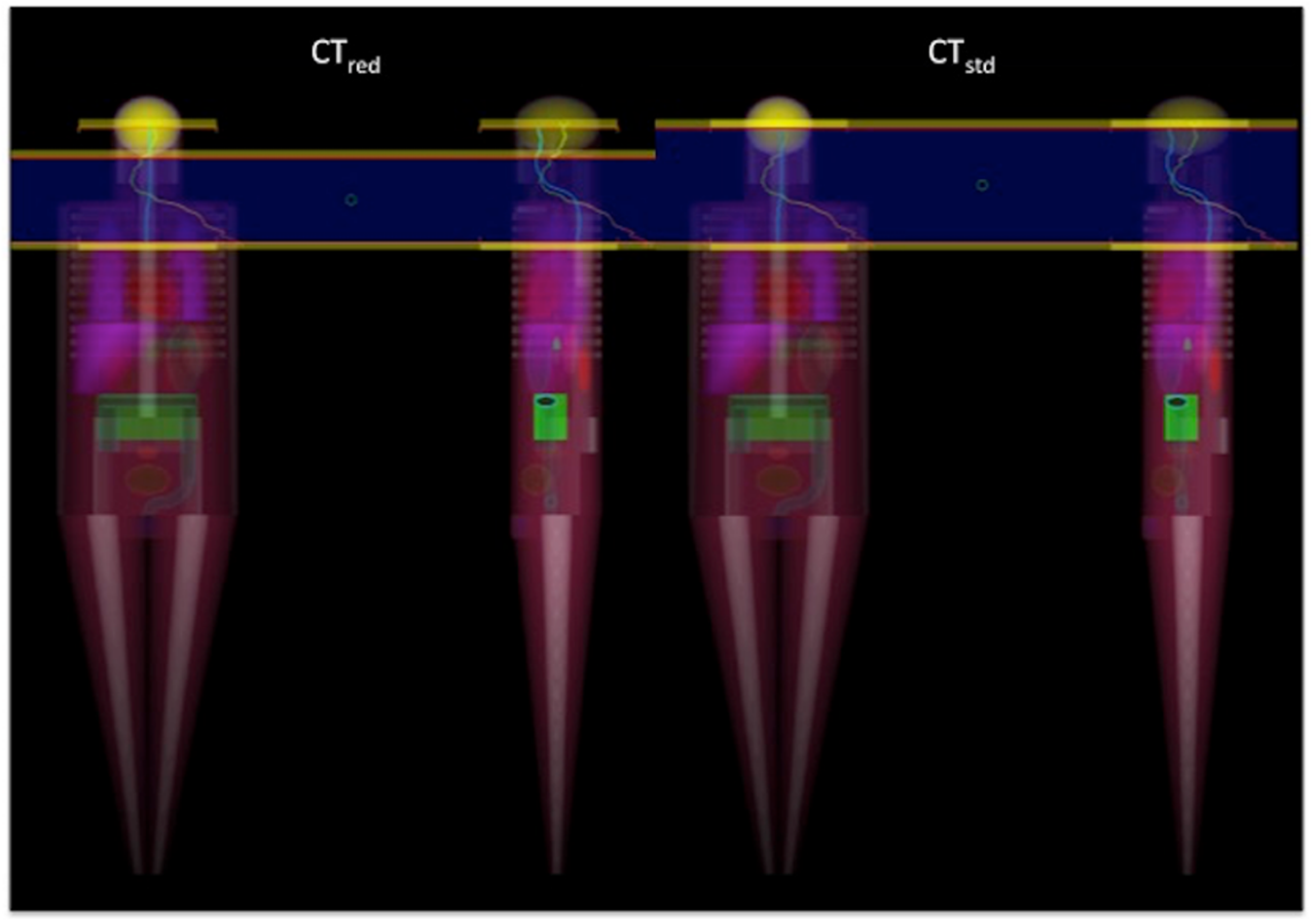
Example for scan range adaptation using the interactive dose-simulator tool in a 35 year-old patient with a peritonsillar abscess.

**Fig 3:**
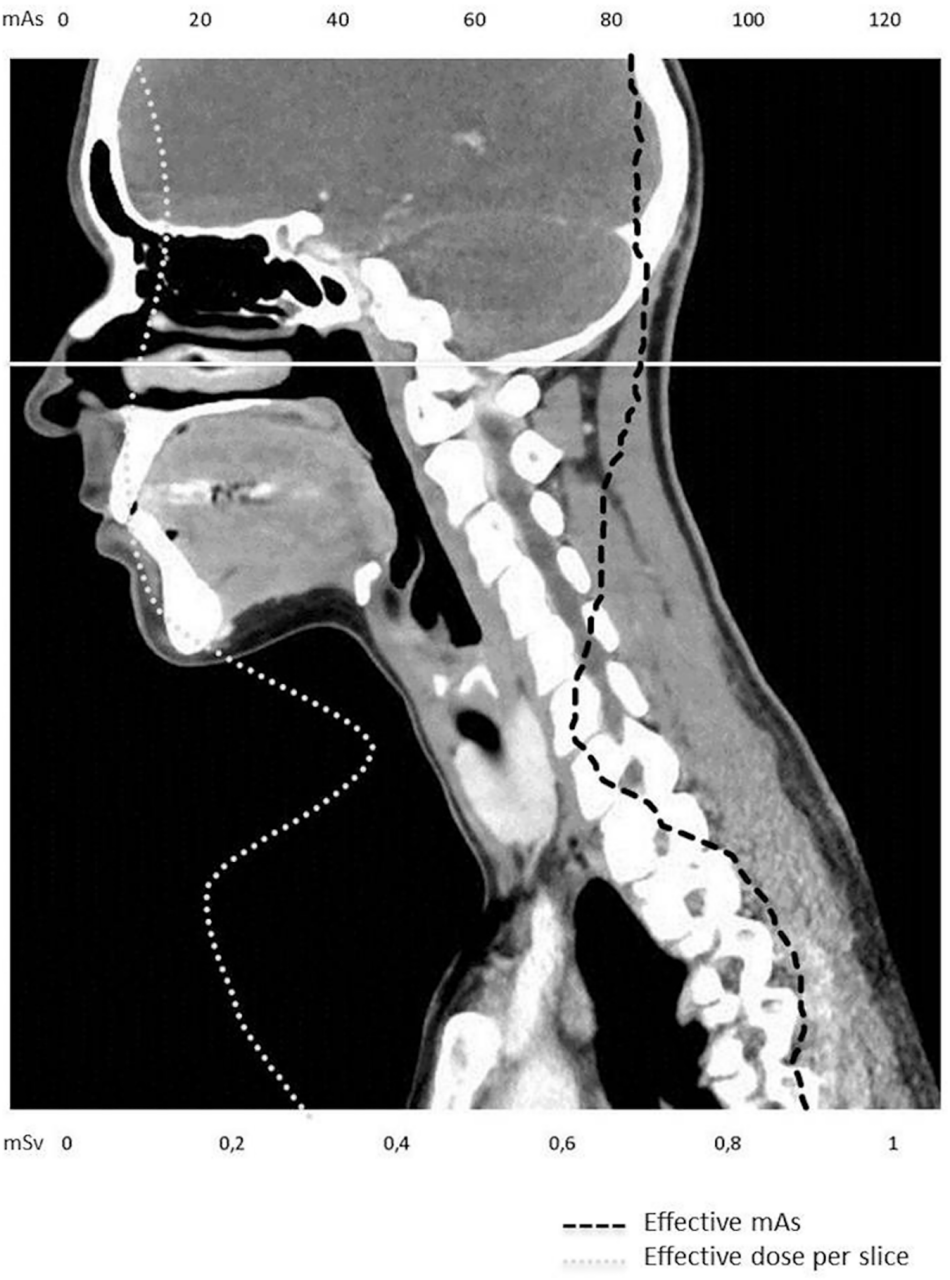
Example of automatic attenuation-based tube current modulation and the resulting slice selective changes of the effective dose as calculated by the dose-simulator tool. The white line indicates the scan range of CT_red_ (reduction of scan length 26%). The DLP could be reduced by 23%, which is approximately similar to the scan range reduction, due to the proportional mathematical relationship. This also applies for the DLP derived effective dose estimation (dose reduction of 23% with an effective dose of 0.7 mSv for CT_red_ and 0.9 mSv for CT_std_.). The dose management platform calculations however revealed an effective dose reduction of only 14% (1.3 mSv for CT_red_ and 1.5 mSv for CT_std_) and the absolute effective dose values are approximately 45% higher than for the DLP derived approach. This can be explained by the fact, that the dose management platform algorithm selectively considers radiosensitive organs in the irradiated area and their individual contribution to the effective dose whereas the DLP derived approach only allows for a rough dose estimation that underestimates the actual effective dose and at the same time overestimates the relative dose reduction associated with the scan range reduction due to the underlying mathematical principles.

Dose measurements were performed with a commercially available, web-based dose management platform (Radimetrics Enterprise Platform, Version 2.6 b, Bayer, Germany). Based on Monte Carlo simulations, this platform allows for an automatic and slice specific calculation of different dose values for arbitrary scan ranges on the basis of a given examination protocol. For both, CT_red_ and CT_std_ effective dose and organ doses (according to the ICRP 103 publication) for relevant organs of interest (brain; breast; esophagus; eye lenses; heart; lungs; red bone marrow; salivary glands) were calculated and compared by subsequently adapting the scan range according to the raw data reconstructions using the interactive dose-simulator tool. Typical scan range adaptation using the interactive dose-simulator tool is shown in Fig 2. Fig 3 indicates the attenuation-based tube current modulation of the CT scan and the resulting slice selective effective dose as calculated by the dose-simulator tool.

#### Conventional dose estimation

To evaluate and compare the performance of the dose management platform to conventional dose calculation approaches, the effective dose was also estimated from the dose length product of CT_red_ and CT_std_ using a scan-region specific conversion factor [20] with the following equation,

Effectivedose=κ×DLP (1) κ = conversion factor = 0.0051

as this is a widely used shortcut method in clinical routine for effective dose determination [21]. In addition to effective dose, size-specific dose estimation (SSDE) was calculated as shown by Christner et al. [17]. In line with previous studies, the measurements of the anterior-posterior and lateral diameter were performed at the height of the fourth cervical vertebral body [11,12].

### Statistical analysis

All statistical analyses were performed using SPSS Statistics (Version 22, IMB, Armonk/NY, USA). Sensitivity and specificity was calculated for both readers independently. Student´s paired t-test with Bonferroni correction was conducted to compare dose values of CT_red_ and CT_std_. P-values ≤ 0.05 were considered to indicate statistical significance.

## Results

**Fig 4:**
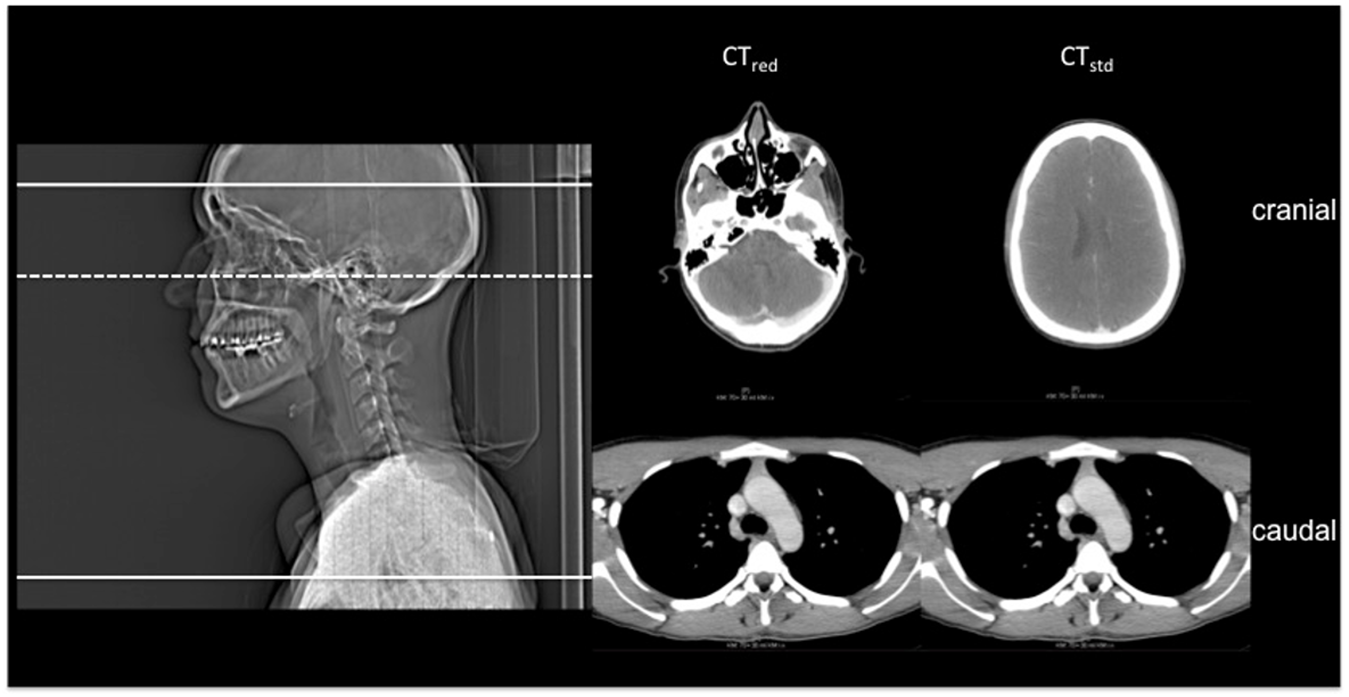
Scout image and the most cranial as well as most caudal slices of CT_red_ and CT_std_ of a 33 year-old patient with a parapharyngeal abscess. The white lines in the scout image indicate the cranial (CT_std_—solid line; CT_red_—dashed line) and caudal (solid line for both, CT_std_ and CT_red_) margin of the scan range. Effective dose could be reduced by approximately 10% (2.2 mSv for CT_std_ and 2.0 mSv for CT_red_).

Contrast-enhanced neck CT was successfully performed in all patients (age 50.2±16.1 [range 22–92]; male 28; female 23) and all examinations were included in the final analysis. The mean scan range could be reduced significantly by approximately 24% for CT_red_ as compared to CT_std_ (21.4±3.0 cm vs. 27.9±3.3 cm, respectively; p<0.001). An image example is provided in Fig 4. Further patient demographics are provided in Table 1.

### Image analysis

**Fig 5:**
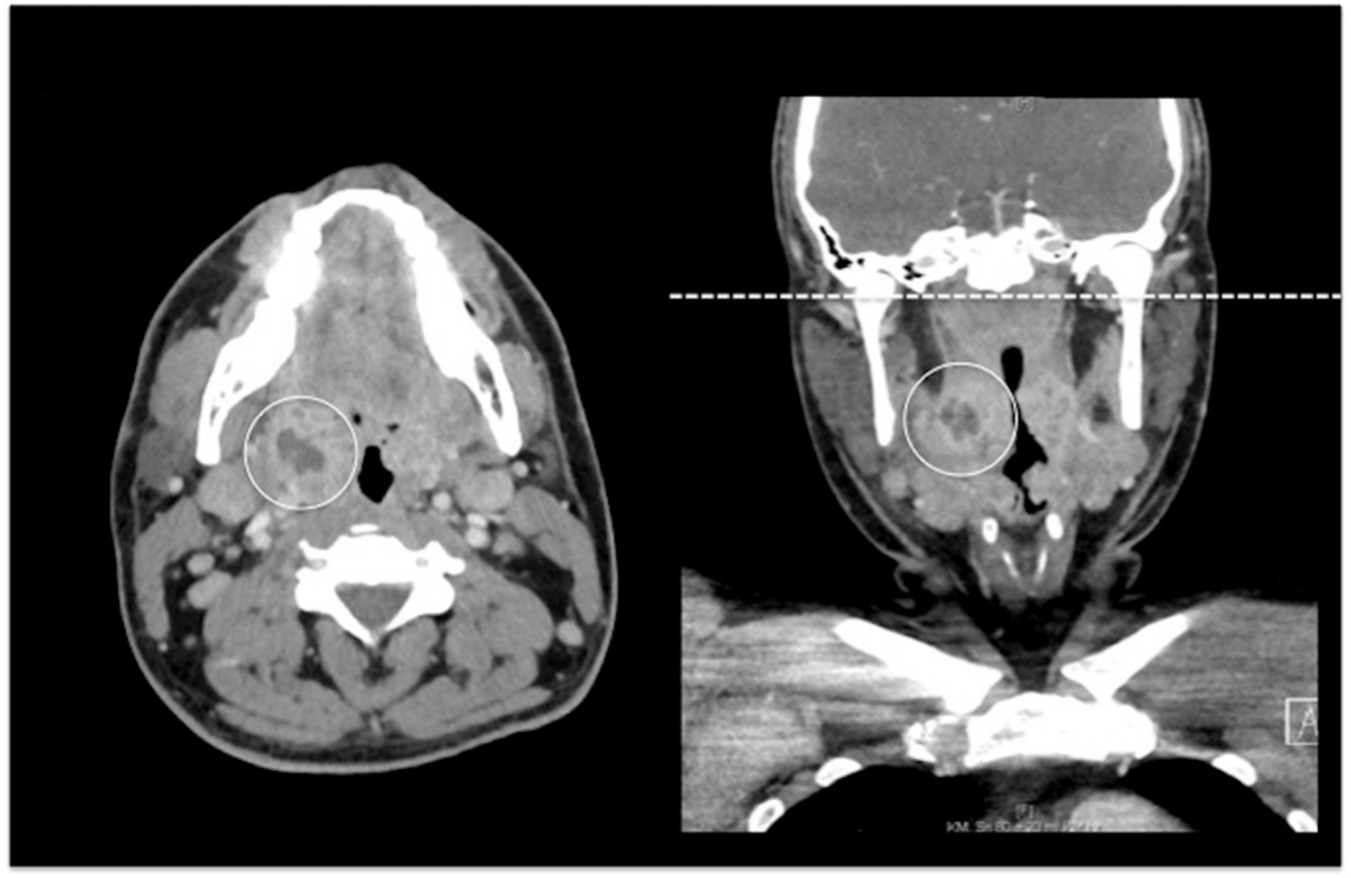
Axial and coronal images of a 31 year-old patient with a peritonsillar abscess (circle). The dashed line indicates the cranial margin of the CT_red_ reconstruction clearly demonstrating that the abscess would have also been covered in its full extent with a reduced scan length without affecting the diagnostic performance. In this case effective dose could be reduced by approximately 14% (4.2 mSv for CT_std_ and 3.6 mSv for CT_red_).

A total of 41 abscesses were identified from both readers in CT_std_. All abscesses could also be detected in their full extent in the CT_red_ reconstructions with a resulting sensitivity and specificity of 1.0 in CT_red_ and CT_std_. For image examples refer to Fig 5.

Three clinically relevant incidental/alternative findings were observed in CT_std_ (unclear thoracic lymphadenopathy (n = 2), mass-like lesion of the proximal esophagus (n = 1)), which were equally detectable in their full extent in the CT_red_ reconstructions.

### Dose measurements

#### Dose management platform

Reduced scan coverage of CT_red_ allowed for a significant reduction of effective dose as compared to the CT_std_ scan (3.5±2.1 mSv and 3.9±2.2 mSv, respectively; p<0.001) by approximately 11%.

Highly significant dose reductions (p<0.001) were also found for relevant organs of interest adjacent to the scan field (brain -84%, eye lenses -90%) as well as in the direct beam (red bone marrow -20%, salivary glands -84%). A detailed overview of all dose calculations is provided in Table 2.

**Table 2:** Descriptive statistics and results of pairwise comparison for effective dose and organ doses after Bonferroni correction.

	CT_red_	CT_std_	t	p
**Effective dose (mSv)**	3.5±2.1	3.9±2.2	-16.4	<0.001
**Organ doses (mSv)**				
• **Brain**	2.3±1.2	14.9±6.6	-16.0	<0.001
• **Breast**	4.2±5.4	4.2±5.4	-3.2	1.00
• **Esophagus**	5.7±3.5	5.8±3.5	-2.2	0.30
• **Eye Lenses**	2.9±1.8	29.0±11.2	-17.3	<0.001
• **Heart**	2.3±2.2	2.3±2.3	-1.3	1.00
• **Lungs**	7.0±4.9	7.1±4.9	-2.0	0.45
• **Red Bone Marrow**	3.9±2.2	4.8±2.5	-11.2	<0.001
• **Salivary Glands**	2.3±1.2	14.9±6.6	-16.2	<0.001

CT_red_ = reduced scan range; CT_std_ = standard scan range; mSV = Millisivert.

#### Conventional dose estimation

Calculation of the DLP also revealed a significant dose reduction for CT_red_ as compared to the standard examination by approximately 22% (309.4 mGy x cm vs. 397.4 mGy x cm, respectively; p<0.001).

The effective dose calculated from the DLP using a scan region specific conversion factor was 1.6 mSv for CT_red_ and 2.0 mSv for CT_std_. Effective dose estimation for this method provided substantially lower dose values (approximately -50%) as compared to the slice specific and organ dose related approach utilized in the dose management platform.

A summary of CTDI_vol_, DLP and SSDE for both examination protocols is provided in Table 3.

**Table 3:** Dose values.

Dose values	SE protocol	DE protocol	SE+DE protocol
**CTDI**_**vol**_	15.0±2.6	6.0±0.4	13.0±4.4
**DLP**_**red**_	355.3±86.8	148.7±22.6	309.4±116.0
**DLP**_**std**_	458.1±106.3	185.2±30.1	397.4±148.4
**SSDE**	31.5±6.5	13.9±7.7	27.0±12.3

SE = single-energy; DE = dual-energy; CTDI_vol_ = computed tomography dose index (mGy); DLP = dose length product (mGy x cm); SSDE = size specific dose estimate.

## Discussion

In this study, we evaluated the effect of a reduced z-axis scan coverage on diagnostic performance and radiation dose in patients with suspected cervical abscesses. Our results indicate that a reduced scan range allows for a significant reduction of effective dose and organ doses at similar diagnostic performance as compared to the standard coverage examination.

These findings are clinically relevant, given the fact that the majority of patients with cervical abscesses are typically of younger age, which increases the demand for dose-effective examination protocols [6]. Current dose saving approaches focus on the latest technical hard- and software developments such as automated modulation of tube current and adaption of tube voltage or iterative image reconstruction algorithms [10,11,22,23]. However, the required hard- and software equipment is not available at all institutions. Another important and CT system-independent parameter with direct influences on radiation dose is the z-axis coverage of the scan [24], because exceeding the adequate scan range for a reliable diagnosis is a well-recognized problem and source of unnecessary radiation [25,26]. Most commonly, routine examination protocols use standardized scan ranges for specific indications, e.g. staging examinations, in order to cover all potentially relevant findings. In contrast, there are also some diseases with relatively typical patient history and clinical symptoms, offering the potential to focus the scan range on a limited region, in which the expected pathology is most likely located. Promising results exploiting this technique have already been described for different indications, such as appendicitis, pulmonary embolism and urolithiasis, showing a significant dose reduction at similar diagnostic performance [13,24,27]. Our results are in line these studies as all cervical abscesses and alternative/incidental findings present in the full range scan could also be detected in the reduced scan range reconstructions at a significantly reduced dose exposure. Thus, limited z-axis coverage seems a feasible approach to substantially reduce the radiation burden without affecting diagnostic performance in patients with suspected cervical abscesses. In particular, this holds true for the eye lenses, as medical radiation-induced cataract development is a well-known complication of head and neck CT imaging, especially if frequent examinations are necessary [28].

In our study, we introduced a mean scan range reduction of approximately 24%, by reducing the scan length in the cranial direction. As recommended by *The American College of Radiology–American Society of Neuroradiology–Society of Pediatric Radiology*, we did not alter the scan length in the caudal direction to ensure reliable diagnosis of potentially life-threatening complications of cervical abscesses in the upper thorax, such as mediastinitis [5]. Although in our study cohort no patient was diagnosed with mediastinitis, since this is a relatively rare complication [29], we still consider it relevant not to shorten the scan range in the caudal direction in favor of patient dose reduction.

Up till now, the most commonly utilized dose indices of CT examinations included the CTDI_vol_ and the DLP, as they are readily available from the patient´s protocol [15]. In our study, we found a significantly reduced DLP for the limited scan reconstruction as compared to CT_std_ with approximately the same percentage change as for the scan range alteration, which indicates the proportional dependency of this dose value to the scan length at a given CTDI_vol_. However size-specific patient characteristics and weighting of radiosensitive organs are only insufficiently included in this approach [16]. For more precise dose estimations with regard to patient size, the concept of SSDE (size-specific dose estimation) has been introduced recently [17]. Nevertheless, this approach also lacks the information of the cumulative effective dose and organ doses, which can only be approximated via conversion factors and with a remaining uncertainty about the actual dose delivered [30]. However, reliable results, especially of organ doses, would be of interest as they offer the most detailed insight of the radiation burden [18]. In this context, workflow integrated dose management platforms may be helpful, to facilitate a quicker and easier overview for detailed changes in dose estimates of different examination protocols and alterations of protocol setting. In our study, the DLP derived effective dose estimation revealed a dose reduction proportional to the scan range reduction (by approximately 21% and 24%, respectively), due to the underlying mathematical relationship. The dose management platform however indicated an effective dose reduction of only 11%, which is approximately 50% lower as compared to the DLP derived estimation approach. This can be explained by the fact, that the dose management platform operates with a slice selective and organ dose based algorithm, which considers not only the scan length but also performs a slice selective weighting of radiosensitive organs in the irradiated area to determine their individual contribution to the effective dose. In contrast, the DLP derived approach only allows for a rough dose estimation that substantially underestimates the actual effective dose and at the same time overestimates the relative dose reduction associated with the scan range reduction, because of the proportional dependency between the scan range and the DLP, as well as between the DLP and the effective dose, without considering radiosensitive organs in the irradiated area. In addition, a reduced z-axis coverage not only allows for a significantly reduced effective dose and reduced organ doses adjacent to the direct beam (brain, eye lenses) but interestingly also in the direct beam itself (e.g. salivary glands). The most likely explanation for this finding is the reduced scatter-radiation due to the limited scan range reconstruction which was not expected and would not have been noticed when evaluating the DLP or the effective dose alone. This indicates the clinical benefit of dose management platforms to assess for relevant changes in protocol settings in order to reduce patient dose burden.

This study has some limitations. First, due to the retrospective study design, no information about the feasibility in clinical routine is available regarding the exact scan range estimation by the operating technician, which needs to be evaluated in further prospective studies. In addition, we only included examinations with activated automatic attenuation-based tube current modulation given the fact, that this is the standard in our institution. Thus, no separate information about the effect of this acquisition technique on the imparted dose is available. Finally, although we did not miss any alternative/incidental findings in our study cohort with the reduced scan range, the number of included patients might have been too small to generalize our findings.

## Conclusions

In conclusion, our findings indicate that reducing z-axis coverage of neck CT allows for a significant reduction of effective dose and organ doses in patients with clinically suspected cervical abscess at similar diagnostic performance as compared to CT_std_.

## Supporting information

S1 Table Dataset including the individual organ doses of CT_red_ and CT_std_ derived from the dose management platform using the interactive dose-simulator tool. (XLSX)
